# Mortality and potential years of life lost by road traffic injuries in Brazil, 2013

**DOI:** 10.1590/S1518-8787.2016050006465

**Published:** 2016-09-22

**Authors:** Silvânia Suely Caribé de Araújo Andrade, Maria Helena Prado de Mello-Jorge

**Affiliations:** I Programa de Pós-Graduação em Saúde Pública. Faculdade de Saúde Pública. Universidade de São Paulo. São Paulo, SP, Brasil; II Departamento de Epidemiologia. Faculdade de Saúde Pública. Universidade de São Paulo. São Paulo, SP, Brasil

**Keywords:** Accidents, Traffic, mortality, Potential Years of Life Lost, Transportation, Hospital Information Systems, Mortality Registries

## Abstract

**OBJECTIVE:**

To estimate the potential years of life lost by road traffic injuries three years after the beginning of the Decade of Action for Traffic Safety.

**METHODS:**

We analyzed the data of the *Sistema de Informações sobre Mortalidade* (SIM – Mortality Information System) related to road traffic injuries, in 2013. We estimated the crude and standardized mortality rates for Brazil and geographic regions. We calculated, for the Country, the proportional mortality according to age groups, education level, race/skin color, and type or quality of the victim while user of the public highway. We estimated the potential years of life lost according to sex.

**RESULTS:**

The mortality rate in 2013 was of 21.0 deaths per 100,000 inhabitants for the Country. The Midwest region presented the highest rate (29.9 deaths per 100,000 inhabitants). Most of the deaths by road traffic injuries took place with males (34.9 deaths per 100,000 males). More than half of the people who have died because of road traffic injuries were of black race/skin color, young adults (24.2%), individuals with low schooling (24.0%), and motorcyclists (28.5%). The mortality rate in the triennium 2011-2013 decreased 4.1%, but increased among motorcyclists. Across the Country, more than a million of potential years of life were lost, in 2013, because of road traffic injuries, especially in the age group of 20 to 29 years.

**CONCLUSIONS:**

The impact of the high mortality rate is of over a million of potential years of life lost by road traffic injuries, especially among adults in productive age (early mortality), in only one year, representing extreme social cost arising from a cause of death that could be prevented. Despite the reduction of mortality by road traffic injuries from 2011 to 2013, the mortality rates increased among motorcyclists.

## RESUMO

**OBJETIVO:**

Estimar os anos potenciais de vida perdidos por acidente de transporte terrestre após três anos do início da Década de Ação pela Segurança no Trânsito.

**MÉTODOS:**

Foram analisados os dados do Sistema de Informações sobre Mortalidade correspondentes aos acidentes de transporte terrestre, em 2013. Foram calculadas as taxas de mortalidade bruta e padronizada para o Brasil e regiões geográficas. Foi calculada, para o País, a mortalidade proporcional segundo faixas etárias, escolaridade, raça/cor da pele e tipo ou qualidade da vítima enquanto usuária da via pública. Foram estimados os anos potenciais de vida perdidos segundo sexo.

**RESULTADOS:**

A taxa de mortalidade, em 2013, foi de 21,0 óbitos por 100 mil habitantes para o País. A região Centro-Oeste apresentou a taxa mais elevada (29,9 óbitos por 100 mil habitantes). A maioria dos óbitos por acidentes de transporte terrestre foi observada no sexo masculino (34,9 óbitos por 100 mil homens). Mais da metade das pessoas que faleceram em decorrência de acidentes de transporte terrestre eram da raça/cor da pele negra, adultos jovens (24,2%), indivíduos com baixa escolaridade (24,0%) e motociclistas (28,5%). A taxa de mortalidade, no triênio 2011 a 2013, apresentou redução de 4,1%, mas aumentou entre os motociclistas. Em todo o País, mais de um milhão de anos potenciais de vida foram perdidos, em 2013, devido aos acidentes de transporte terrestre, especialmente na faixa etária de 20 a 29 anos.

**CONCLUSÕES:**

O impacto da alta taxa de mortalidade é de mais de um milhão de anos potenciais de vida perdidos por acidentes de transporte terrestre, principalmente entre adultos em idade produtiva (mortalidade precoce), em apenas um ano, representando extremo custo social decorrente de uma causa de óbito que poderia ser prevenida. Apesar da redução da mortalidade por acidentes de transporte terrestre de 2011 a 2013, as taxas de mortalidade aumentaram entre os motociclistas.

## INTRODUCTION

The annual number of deaths resulting from road traffic injuries (RTI) estimated by the World Health Organization (WHO) is 1.24 million people, mostly living in middle-income countries^a^. The RTI are among the 10 leading causes of death in the world according to data from the WHO for 2012^b^. In 2007, RTI deaths accounted for 29.3% of mortality by external causes and, along with murders, account for about 2/3 of the external causes deaths in Brazil^13^.

In Brazil, considering the period from 1998 – year of implantation of the Brazilian Traffic Code – to 2008, the number of deaths by RTI increased 121.0% (3,736 and 8,093 deaths, respectively). The mortality rate by RTI in the Country increased between the years 2000 and 2010, ranging from 18 to 22.5 deaths per 100,000 inhabitants^11^. Alcohol consumption associated with driving is one of the main risk factors for death by RTI, as well as being male and young adult^2^ ^,^ ^13^. Specifically, alcohol is responsible for about one third of the deaths in traffic, being the most important determinant for the severity of injuries and fatality of accidents^9^.

Data from the Surveillance System of Risk and Protection Factors for Chronic Diseases by Telephone Survey (VIGITEL), between 2007 and 2013, showed a trend of reduction in the habit of ingesting alcohol and driving among adults in Brazilian state capitals and in the Federal District after legislative frameworks restricting this action^5^. This highlights the importance of public policies that, according to Mello-Jorge and Adura, defend the society and place the collective before the individual interest^9^.

Given the concern about the magnitude of mortality by RTI, the United Nations General Assembly of 2009, by a resolution, set the period from 2011 to 2020 as the Decade of Action for Traffic Safety. The Assembly encouraged the member-countries to create an action plan to reduce the number of deaths and non-fatal victims by traffic injuries. This plan must involve the governmental and private sectors, as well as society as a whole^c^.

Brazil, in 2010, appointed an inter-ministerial Committee to coordinate the implementation of the Life in Traffic Project^d^, strategy funded by the Bloomberg Philanthropies Foundation and coordinated by the Brazilian Ministry of Health. Alcohol consumption and speeding are two priority axes of activity of the project that was implemented in five Brazilian capitals^4^. The Life in Traffic Project presented impact in reducing the mortality ratio by vehicles and the mortality rate^12^.

Despite the efforts, in 2010, the RTI were the second most common cause of death in the Country^e^. One of the ways to assess the impact of mortality by RTI to society is by measuring the potential years of life lost (PYLL) by incapacity arising from this harm; “since this a way to see the influence of accidents on life expectancy, revaluing deaths from this cause because of the fact that they occurred in early stages of life”^f^.

The aim of this study was to estimate the potential years of life lost by road traffic injury three years after the beginning of the Decade of Action for Traffic Safety.

## METHODS

We conducted a descriptive study of mortality rates by road traffic injuries in Brazil in the years 2011, 2012, and 2013. We selected the codes for the deaths by place of residence according to the tenth revision of the International Classification of Diseases (ICD-10)^21^, corresponding to road traffic injuries: categories V00 to V89 of the chapter XX (External Causes of Morbidity and Mortality), grouped according to type of victim (according to ICD-10: pedestrians, cyclists, motorcyclists [including tricycles], car occupant [including pickup truck], heavy transport occupant [truck], bus occupant, and others [other means of ground transportation or cases in which the victims were not specified]).

We estimated the mortality rates using the Mortality Information System (SIM) as data source^g^. As denominator of the rates analyzed, we used the estimated population for Brazil and regions in the years 2011, 2012, and 2013, obtained from the Internet address of the Brazilian Institute of Geography and Statistics^h^.

For comparison between the regions, only in the year 2013, we chose to standardize, by the direct method, the regional mortality rates for the Brazilian population in 2013. We estimated the mortality rates according to sex (male; female) and the proportional mortality by age group, education level, race/skin color, and type of victim for Brazil in 2013.

We estimated the PYLL according to sex, adapting the method proposed by Romeder and McWhinnie^15^ ^,^ ^16^ regarding the upper limit of age. After the exclusion of deaths occurred in individuals under the age of one year old and over 70 years old, we proceeded to estimate midpoint of age groups. Thus, we subtracted from 70 years (proposed age in the original method^15^ ^,^ ^16^) the midpoint of each age group and multiplied it by the number of deaths in each age group.

These totals were added, and we obtained the total PYLL. Deaths due to RTI that occurred before 70 years old set the precocity of them^10^. PYLL rates were estimated by dividing the PYLL in each age group by the corresponding population, multiplied by 100,000 inhabitants. We estimated the average of PYLL per total death (PYLL/death), according to sex and age group.

## RESULTS

In 2013, 42,266 deaths occurred throughout the Brazilian territory by RTI, designing a mortality rate of 21.0 deaths per 100,000 inhabitants, greater in males (34.9 deaths per 100,000 males). The proportional mortality by RTI was higher in the age group of 20 to 29 years, followed by individuals between 30 and 39 years (Table 1).

**Table 1:** Distribution of deaths by Road Traffic Injuries and description of the proportional mortality according to type of victim and sociodemographic characteristics. Brazil, 2013.

Variable	n	Proportional mortality (%)
Sex		
Male	34,629	82.0
Female	7,617	18.0
Age group (years)		
< 1	112	0.3
1-4	461	1.1
5-9	497	1.2
10-14	736	1.7
15-19	3,425	8.1
20-29	10,207	24.2
30-39	8,357	19.8
40-49	6,764	16.0
50-59	5,040	11.9
60-69	3,377	7.9
70-79	2,101	4.9
≥ 80	1,013	2.4
Ignored age	288	0.7
Education level (years of schooling)		
None	2,082	4.9
1-3	6,767	16.0
4-7	10,159	24.0
8-11	9,315	22.0
≥ 12	2,407	5.7
Ignored	11,536	27.3
Race/skin color		
White	18,025	42.7
Black	22,325	52.8
Yellow	121	0.3
Indigenous	100	0.2
Ignored	1,695	4.0
Type of victim		
Pedestrian	8,220	19.4
Cyclist	1,348	3.2
Motorcyclist	12,040	28.5
Car occupant	10,084	23.9
Heavy transport occupant	818	1.9
Bus occupant	173	0.4
Other and unspecified	9,583	22.7

Total	42,266	100

Regarding education, the proportional mortality was higher among those with four to seven years of schooling (24.0%), even when the analysis was conducted only among adults over 20 years old (23.6%). More than 1/4 of death cases by RTI were classified in the SIM database as ignored information for the education variable (Table 1). The percentage of ignored information about deaths arising from RTI according to race/skin color was 4.0%. Black race/skin color represented 52.8% of deaths by RTI in 2013. By type of victim, the deaths occurred more often among motorcyclists and car occupants (Table 1).

The crude mortality rate by RTI was higher in the Midwest region, followed by the Southern region. Regarding the standard rates for the Brazilian population of the year of the study, the Midwest and Northeast regions presented the highest rates (Table 2).

**Table 2:** Number of deaths and crude and standardized mortality rates (per 100,000 inhabitants) by road traffic injuries. Brazil and geographic regions, 2013.

Variable	n	Mortality rate*	
	
Region		Crude	Standardized
North	3,446	20.3	22.4
Northeast	12,665	22.7	23.5
Southeast	14,707	17.4	16.8
South	6,960	24.2	23.4
Midwest	4,488	29.9	30.0

Brazil	42,266	21.0	21.0

* per 100,000 inhabitants.

Comparing the early years of the Decade of Action for Traffic Safety, the mortality rates by RTI ranged from 21.9 to 21.0 deaths per 100,000 inhabitants in 2011 and 2013, respectively. The mortality rate by RTI decreased 4.1% in the analyzed period. Among motorcyclists, the mortality by RTI increased (+1.7%) from 2011 to 2013. Among pedestrians, the 2013 mortality rate by RTI decreased (-12.8%) in relation to 2011 (Table 3).

**Table 3:** Number of deaths and crude and standardized mortality rates (per 100,000 inhabitants) by road traffic injuries according to type of victim. Brazil, 2011 to 2013.

Type of victim	2011		2012		2013	
		
	n	Rate*	n	Rate*	n	Rate*
Pedestrian	9,244	4.7	8,819	4.4	8,220	4.1
Cyclist	1,475	0.8	1,492	0.8	1,348	0.7
Motorcyclist	11,485	5.8	12,544	6.3	12,040	5.9
Car occupant	10,112	5.1	10,525	5.3	10,084	5.0
Heavy transport occupant	848	0.4	863	0.4	818	0.4
Bus occupant	194	0.1	193	0.1	173	0.1
Other and unspecified	9,898	5.0	10,376	5.2	9,583	4.8

Total	43,256	21.9	44,812	22.5	42,266	21.0

* per 100,000 inhabitants.

There were 1,309,191.5 years of potential life lost in 2013. The PYLL rate was of 253,831 days (694.5 years) lost for every 100,000 inhabitants. The proportion of lost years by RTI was, on average, 33.8 years per death registered in 2013. The age group of 20 to 29 years presented the highest proportion of PYLL due to RTI in both sexes. We observed greater loss of days of life among men than among women (Table 4).

**Table 4:** Potential years of life lost, percentage, rate, and mean by road traffic injuries, according to sex and age. Brazil, 2013.

Age group (years)	Total				Male				Female			
		
	PYLL	%	PYLL Rate	Mean*	PYLL	%	PYLL Rate	Mean*	PYLL	%	PYLL Rate	Mean*
1-4	23,557.5	1.8	193.2	67,5	12,892.5	1.2	206.8	67,5	10,665.0	4.8	178.9	67,5
5-9	31,311.0	2.4	192.8	63.0	19,467.0	1.8	234.7	63.0	11,844.0	5.3	149.1	63.0
10-14	42,688.0	3.3	248.9	58.0	26,912.0	2.5	308.0	58.0	15,776.0	7.1	187.7	58.0
15-19	181,525.0	13.9	1,058.9	53.0	146,492.0	13.5	1,682.7	53.0	35,033.0	15.7	415.3	53.0
20-29	464,373.0	35.5	1,341.2	45.5	400,855.0	36.9	2,297.8	45.5	63,518.0	28.4	369.8	45.5
30-39	296,602.5	22.7	918.3	35.5	255,209.5	23.5	1,585.8	35.5	41,393.0	18.5	255.4	35.5
40-49	172,456.5	13.2	662.5	25.5	145,248.0	13.4	1,136.0	25.5	27,208.5	12.2	205.4	25.5
50-59	78,104.5	5.9	384.2	15.5	64,371.5	5.9	658.6	15.5	13,733.0	6.2	130.1	15.5
60-69	18,573.5	1.4	148.4	5.5	14,355	1.3	247.7	5.5	4,218.5	1.9	62.8	5.5

Total	1,309,191.5	100	694.5	33.8	1,085,802.5	100	1,156.8	33.7	223,389	100	236.0	34.1

PYLL: potential years of life lost; PYLL: potential years of life lost per 100,000

* Average per 10,000 inhabitants.

## DISCUSSION

In this study, most deaths by RTI were observed in males, among young adults (20 to 39 years of age). According to WHO data, over 3/4 of deaths by RTI in the world are of young men^a^. Brazilian males also die more by RTI than women^1^ ^,^ ^2^ ^,^ ^10^ ^,^ ^11^, and the mortality trend by RTI among men in Brazil increased in the period from 1998 to 2007^3^. A study conducted on the Federal District described the deaths by injuries with motorcyclists from 1996 to 2007 and found that the profile of the injured ones was male (94.3%), brown (71.0%), and aged between 20 and 39 years (73.8%)^10^.

Here, mortality by RTI was higher among people with low education level, with prevalence among individuals with four to seven years of schooling. Schooling and race/skin color variables showed high (> 20.0%) and very low (< 5,0%) percentages of ignored data, respectively. However, the improvement of SIM data quality is noticeable, with gradual reduction of the ignored percentage^8^. The proportional mortality by RTI was more pronounced among individuals with low schooling and black individuals, indicating socioracial inequalities in mortality and exposure to risk factors for this harm.

Pointing out that alcohol consumption is one of the risk factors for RTI, a study based on data from the Surveillance System of Violence and Accidents held in 2011 identified that, among the victims met in emergency services due to injuries, the prevalence of self-reported alcohol consumption was significantly higher among individuals with low education – zero to eight years of schooling – (Prevalence Ratio [PR] = 1.26; 95%CI 1.16–1.37) and black individuals (PR = 1.54; 95%CI 1.35–1.76) than among white people^7^.

Regarding race/skin color, more than half of the individuals who have died due to RTI were black. A research on the trend of mortality in Brazil between the years 2000 and 2010 using SIM data (with correction for sub-record of deaths and poorly defined causes) observed that the black population showed the highest percentages of mortality from external causes (17.7% in 2000 and 17.6% in 2010). For the white population, the percentages were of 10.7% and less than 1.0% in 2000 and 2010, respectively^e^.

In this study, the higher rates of mortality by RTI have been found in the Midwest region. A study that investigated the trends in the risk of death by RTI in men between 20 and 49 years of age, between 1980 and 2005, points out that this risk was 1.3 times higher in the capitals of the North and Midwest regions than in all the capitals of the Country. The trend of the risk of death by RTI in the studied group was of stability; however, after the implementation of the Brazilian Traffic Code in 1998, the risk decreased^f^.

An analysis of the trend of mortality by motorcycle accidents in the period from 1996 to 2009 concluded that the highest growth rates of mortality were in the North, Northeast, and Midwest regions^6^. Other time series analysis from 1991 to 2007 about RTI mortality showed that the highest rates were from the Midwest and North regions, and superior to the national ones^14^. This increase can be attributed to the increased fleet of vehicles, alcohol consumption and driving, low proportion in the use of safety equipment, and deficiency in medical and hospital care in the states belonging to these geographical regions^5^ ^-^ ^7^ ^,^ ^i^.

The increased mortality occurred in all external causes, since there was a tendency of increase in mortality due to accidents and violence, between the years 2000 and 2010, in the North, Northeast, South, and Midwest regions, considering the data corrected for poorly defined causes and sub-records of deaths^3^. We point out that these data refer to all external causes and not to the RTI specifically.

Comparing the years 2011 to 2013, we observed reduction in mortality rates by RTI, as stated in other studies in the Country during the periods from 1980 to 2003^18^ and 1996 to 2007^14^. However, there is other evidence that shows the growth of 22.5% in the mortality rates by RTI between the years 2000 and 2010^i^.

By type of victim, motorcyclists showed both higher proportional mortality in 2013 and higher mortality rates in the triennium 2011-2013. The literature shows high mortality rates among motorcyclists and their worrisome growth over time^14^ ^,^ ^18^. We observed decrease in mortality among pedestrians in the studied period, corroborating the reduction tendency presented by other studies^2^ ^,^ ^11^. As likely explanatory factor is the greater dissemination of traffic education topics, including the use of mass media and the emphasis on using the crosswalk.

The lowest death rates were observed in the Southeast region in the year 2013, corroborating the data of Souza et al.^18^ (2007), which showed a more pronounced declining trend in the RTI mortality rates in that region. This can be attributed to the better implementation of the Brazilian Traffic Code in this region because of cultural factors related to the oldest history with issues involving traffic^18^.

Thus, there might have occurred increase or decrease of the considered rates according to periods and geographic regions. The increase in the number of motorcycles, whether as work or transportation vehicle, may have influenced the increased mortality rates by RTI in Brazil. The reasons for the increase in the fleet of motorcyclists can be attributed to the precariousness of public transportation, phone-delivery services, possibility of work for young people, and the ease of buying a motorcycle^2^.

According to a study on global disease burden conducted by the World Health Organization, RTI were the tenth cause (9,600,000 of PYLL) among the main diseases and harms with the higher number of years of life lost in 1990 in the world. In 2010, the RTI reached the fifth position among the 50 leading causes of premature mortality, with total loss of 5,900,000 of PYLL^4^.

The findings of this study on PYLL showed higher values than those found in other works using similar method, showing that RTI have higher impact on premature mortality in the Brazilian society. The PYLL by leptospirosis year 2007 were estimated at 6,490, with 75.0% of these in the age group of 20 to 49 years^19^. For hepatitis B, also in Brazil, 9,353 potential years of life were lost during the year of 2009, mostly in the age group of 40 to 49 years^20^.

An analysis carried out with data from the state and city of Rio de Janeiro regarding the year 1990 identified loss of 107,687.5 and 48,015.0 years of life by RTI, respectively^13^. In Pernambuco, in 2007, 48,006 years of life were lost by RTI, and the age group with the highest PYLL rate was 20 to 39 years, regardless of the type of victim. Males had the highest number of PYLL: 41,027^1^.

Another study, conducted in the state of Santa Catarina from 1998 to 2000, found loss of 201,879.5 years of life by RTI among individuals from zero to 80 years old. From these, 78.0% were male and 31.3% were in the age group of 20 to 29 years^f^. We observed in this study that the age group in which there has been greater loss of potential years of life comprehended the ages from 20 to 59 years. This group of individuals was in economically active age, representing not only individual and family impact, but also collective impact because of the termination of their economic and intellectual potential^16^.

The exclusion of deaths of individuals under the age of one year old and over 70 years old in the calculation of PYLL may cause underestimation of this indicator. Another limitation is that deaths by RTI may be underestimated in the SIM database, because there is a considerable component of external causes of unspecified type (undetermined intent)^c^, which can hide deaths by RTI.

Most of the traffic injuries or accidents are preventable^i^. According to Souza et al.^17^, RTI should be considered as violence, as they result from omissions and violations both from the government and involved ones in accidents, destituted, thus, from the character of eventuality or fatality^18^. In short, “death is the ultimate expression of the problem of violence in a society”^3^.

In 2001, by the National Policy of Reduction of Morbidity and Mortality by Accidents and Violence, Brazil assumed that these harms are effectively public health problems and set out the specific actions of the health sector in the field of surveillance, health promotion, injury prevention, care, recovery, and rehabilitation^i^. However, the approach regarding RTI must overcome the obsolete man-vehicle-lane triad^18^; the interventions directed to the RTI, because of their multiple causes, need to be multidisciplinary and intersectoral, as well as count on the effective community participation^i^.

The Ministry of Health develops several actions for the fight against traffic violence. Among them are the monitoring of violence and accidents by the survey component of the Surveillance System of Violence and Accidents implemented in 2006, and the Life in Traffic Project, operating in the two main risk factors for the RTI in five Brazilian capitals by the classification of information, planning, monitoring, follow-up, and evaluation of interventions. However, we need articulated and intersectoral actions involving all society, aiming at the fulfilment of the goals of reducing morbidity and mortality by RTI set in the Decade of Action for Traffic Safety from 2011 to 2020^c^.

The impact of more than a million PYLL by RTI, especially in young and productive age (early mortality), in only one year, in the Country, represents an extreme social cost arising from a cause of death that could be prevented. Thus, we need major advances in prevention of mortality by RTI, considering the complexity of factors involved and the uneven, and certainly iniquitous, distribution of this harm in the Brazilian population.

## Footnotes

a World Health Organization. Global status report on road safety 2013: supporting a decade of action. Geneva; 2013. Available from: http://www.un.org/en/roadsafety/pdf/roadsafety2013_eng.pdf

b World Health Organization. The top ten causes of death: the ten leading causes of death in the world, 2000 and 2012. Geneva; 2012 [cited 2015 May 13]. Available from: http://www.who.int/mediacentre/factsheets/fs310/en/

c Organización de las Naciones Unidas. Resolución aprobada por la Asamblea General de 2 de marzo de 2010. Mejoramiento de la seguridad vial en el mundo - 64/255. [cited 2012 Apr 30]. Available from: http://www.who.int/violence_injury_prevention/publications/road_traffic/UN_GA_resolution-54-255-es.pdf

d Ministério da Saúde. Portaria nº 153 de 11 de agosto de 2010. Institui a Comissão Nacional Interministerial para acompanhamento da implantação e implementação do Projeto Vida no Trânsito. *Diario Oficial Uniao*. 11 ago 2010; Seção 1:79.

e Ministério da Saúde, Secretaria de Vigilância em Saúde, Departamento de Análise de Situação de Saúde. Saúde Brasil 2011: uma análise da situação de saúde e a vigilância da saúde da mulher. Brasília (DF): Editora do Ministério da Saúde; 2012. Como morrem os brasileiros: tendências e desigualdades nas regiões, unidades federadas e nas categorias de raça-cor nos anos de 2000-2010; p.107-62. Available from: http://bvsms.saude.gov.br/bvs/publicacoes/saude_brasil_2011.pdf

f Henrique MC. Anos potenciais de vida perdidos: a herança dos acidentes de trânsito para as gerações futuras: uma abordagem interdisciplinar [these]. Florianópolis (SC): Universidade Federal de Santa Catarina; 2002.

g Ministério da Saúde. DATASUS. Sistema de Informações de Mortalidade - SIM. [cited 12 May 2015]. Available from: http://www2.datasus.gov.br/DATASUS/index.php?area=060701

h Instituto Brasileiro de Geografia e Estatística. Estimativas populacionais para os municípios brasileiros em 01.07.2013. Rio de Janeiro; 2013 [cited 2015 May 12]. Available from: http://www.ibge.gov.br/home/estatistica/populacao/estimativa2013/

i Ministério da Saúde, Secretaria de Vigilância em Saúde, Departamento de Análise de Situação de Saúde. Política Nacional de Redução de Morbimortalidade por Acidentes e Violências: Portaria MS/GM nº 737 de 16/5/01. Publicada no DOU nº 96, Seção 1E, de 18/5/01. 2.ed. Brasília (DF); 2005. (Série E. Legislação de Saúde). Available from: http://bvsms.saude.gov.br/bvs/publicacoes/politica_reducao_morbimortalidade_acidentes_2ed
